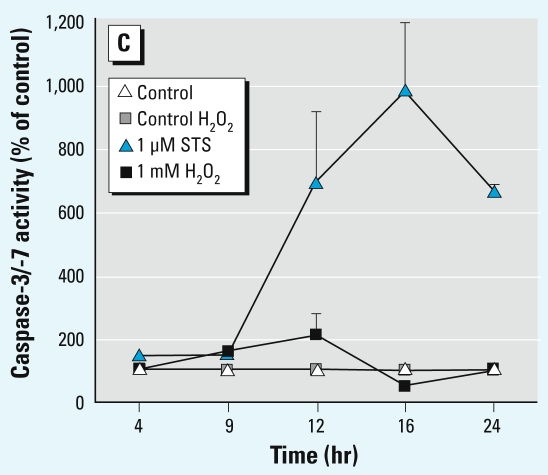# Errata

**Published:** 2009-08

In the June Science Selection article “Prenatal Preview: Early Bisphenol A Exposure May Spawn Late-Life Reproductive Problems” [Environ Health Perspect 117:A256 (2009)], diethylstilbestrol is incorrectly identified as an antinausea drug. This drug was actually used to prevent miscarriage. *EHP* regrets the error.

In the “Discussion” (paragraph 14, p. 701) of the article by Guidotti et al. [Environ Health Perspect 115:695–701 (2007)], the first two sentences (“There appears to have been no identifiable public health impact from the elevation of lead in drinking water in Washington, DC, in 2003 and 2004. This may reflect effective measures to protect the residents, as 153 reported compliance with recommendations to filter their drinking water.”) should have been replaced with the following sentence: “Measures to protect residents from exposure to lead in drinking water may have prevented more frequent elevations in blood lead.” In addition, on page 695 in the right-hand column, line 4, the year 2002 should be given as 2000. The authors apologize for these errors.

In Figure 6C of Moors et al. [Environ Health Perspect 117:1131–1138 (2009)], the symbols for control and 1 μm STS were not correct. The corrected figure is presented here. *EHP* regrets the error.

## Figures and Tables

**Figure:**